# Supplementary material for: Residual Tumor Resection After Anti-PD-1 Therapy: A Promising Treatment Strategy for Overcoming Immune Evasive Phenotype Induced by Anti-PD-1 Therapy in Gastric Cancer
Source: Cells. 2025 Aug 6;14(15):1212. doi: 10.3390/cells14151212 (PMC12346003; doi:10.3390/cells14151212)
Supplement: Supplementary file 1 [file cells-14-01212-s001.zip › cells-3768041-supplementary.pdf]

Supplementary Table S1. The list of antibodies for IHC staining

Antibodies	Company	Catalog number	Source	Clone	Antigen retrival	Dilution
HLA class I	abcam	ab70328	Mouse	EMR8-5	pH 6 for 10 min at 100℃	1:2000
CD8	Agilent Tecchnologies, Inc	M7103	Mouse	C8/144B	pH 9 for 10 min at 100℃	1:200
TIM-3	Cell Signaling Technology	#45208	Rabbit	D5D5R	pH 9 for 10 min at 100℃	1:400
TIGIT	Cell Signaling Technology	#99567	Rabbit	E5Y1W	45 min at 98℃	1:400
PD-L1	Cell Signaling Technology	#13684	Rabbit	E1L3N	pH 9 for 10 min at 105℃	1:400
PD-L2	Cell Signaling Technology	#82723	Rabbit	D7U8C	pH 9 for 10 min at 105℃	1:200
CEACAM-1	abcam	ab243889	Rabbit	BLR032F	pH 6 for 10 min at 100℃	1:500
CD155	Cell Signaling Technology	#81254	Rabbit	D8A5G	-	1:200
MHC class II	Cell Signaling Technology	#68258	Mouse	LGII-612.14	-	1:1000
p-Smad3	abcam	ab52864	Rabbit	EP823Y	pH 9 for 10 min at 100℃	1:200

IHC, immunohistochemical; CEACAM-1, carcinoembryonic antigen-related adhesion molecule-1; HLA, human leukocyte antigen; MHC, major histocompatibility complex; PD-L1, programmed death ligand 1; p-Smad3, phospho-Smad3; TIGIT, T cell immunoglobulin and ITIM domain; TIM-3, T cell immunoglobulin and mucin domain -3.

Supplementary Table S2. Non-curative factors of 97 patients.

Non-curative factors	All patients (*n*=97)	Non-TR group* (*n*=82)	TR group* (*n*=15)	*p*-value
Liver metastasis	26 (26.8%)	21 (25.6%)	5 (33.3%)	0.538
Peritoneal metastasis	39 (40.2%)	34 (41.5%)	5 (33.3%)	0.555
Distant lymph node metastasis	31 (32.0%)	28 (34.1%)	3 (20.0%)	0.374
Lung metastasis	7 (7.2%)	7 (8.5%)	0 (0%)	0.591
Bone metastasis	5 (5.2%)	5 (6.1%)	0 (0%)	>0.999
Positive for peritoneal cytology	15 (15.5%)	12 (14.6%)	3 (20.0%)	0.697
Tumor invasion of adjacent structures	8 (8.3%)	7 (8.5%)	1 (7.1%)	>0.999

*Patients underwent tumor resection (TR) after chemotherapy or anti-PD-1 therapy (TR group) and those underwent chemotherapy and/or anti-PD-1 therapy alone (Non-TR group). TR, tumor resection.

Supplementary Table S3. Non-curative factors of 15 patients who underwent TR after chemotherapy or anti-PD-1 therapy

Non-curative factors	All patients (*n*=15)	Chemo+TR group (*n*=9)	Anti-PD-1+TR group (*n*=6)	*p*-value
Liver metastasis	5 (33.3%)	3 (33.3%)	2 (33.3%)	>0.999
Peritoneal metastasis	5 (33.3%)	3 (33.3%)	2 (33.3%)	>0.999
Distant lymph node metastasis	3 (20.0%)	0 (0%)	3 (50.0%)	0.044
Positive for peritoneal cytology	3 (20.0%)	2 (22.2%)	1 (16.7%)	>0.999
Tumor invasion of adjacent structures	1 (6.7%)	1 (11.1%)	0 (0%)	>0.999

Anti-PD-1+TR, tumor resection after anti-PD-1 therapy; Chemo+TR, tumor resection after chemotherapy; TR, tumor resection.

Supplementary Table S4. The expression of TIGIT on lymphocytes in each case

Chemo+TR group		Anti-PD-1+TR group	
Case		Case	
1	−	8	+
2	−	9	−
3	−	11	+
4	−	13	−
5	−	15	−
6	−		
14	−		

TIGIT was considered positive when there were lymphocytes with membranous staining. Anti-PD-1+TR, tumor resection after anti-PD-1 therapy; Chemo+TR, tumor resection after chemotherapy.
